# The contribution of vibrational spectroscopy and data analytics towards the achievement of the global sustainability goals

**DOI:** 10.1002/jsfa.70206

**Published:** 2025-09-15

**Authors:** Daniel Cozzolino, Louwrens C Hoffman

**Affiliations:** ^1^ The University of Queensland, Centre for Nutrition and Food Sciences (CNAFS), Queensland Alliance for Agriculture and Food Innovation (QAAFI) Brisbane QLD Australia

**Keywords:** Sustainable Development Goals, vibrational spectroscopy, data analytics, artificial intelligence

## Abstract

The United Nations Sustainable Development Goals (UN SDGs) were defined to improve the quality of life of the global population particularly regarding social and economic aspects, with a major focus on environmental sustainability. The incorporation of digital technologies into the agri‐food sector has become a key enabler in increasing the efficiency, productivity, and sustainability of food production and processing systems. Digital technologies and innovations including artificial intelligence (AI), robotics, in‐ground and remote sensors, connectivity, and internet of things (IoT) have been recognized as also being critical for the successful implementation of the UN SDGs. In particular, the utilization of sensing technologies (e.g*.*, in‐ground and remote sensors) has been shown to be of great importance to achieve these goals. The use of vibrational spectroscopy and data analytics have shown potential to contribute with the development and implementation of UN SDGs. An overview of the contribution of sensing technologies based on the utilization of vibrational spectroscopy (e.g*.*, near‐ and mid‐infrared spectroscopy, hyperspectral imaging) and data analytics to achieve the UN SDGs is provided. Advantages and limitations of these techniques are also discussed. The incorporation of technology will provide tools that can be used to monitor and predict the safety and quality of foods. Furthermore, digital technologies are enabling the development of novel decision‐management systems along the food supply and value chain. Ultimately, the goal will be to assure the consumers the use of these technologies plays a key role in the applicability of the UN SDG. © 2025 The Author(s). *Journal of the Science of Food and Agriculture* published by John Wiley & Sons Ltd on behalf of Society of Chemical Industry.

## INTRODUCTION

Current developments combined with the implementation of digital technologies are determining a technological revolution that is contributing to the redesign of several aspects of the economy and society.^1^, ^2^ Several innovative digital interventions are occurring and contributing to disrupt both agriculture (e.g., farm production practices, soil and management) as well as the food supply and value chains (e.g., increase efficiency in food systems, methods to detect and monitor food fraud, etc.).^1^, ^2^ Specifically, these disruptions have determined fundamental changes in agricultural and food production, food safety and security, contributing towards the progress and uptake of the United Nations (UN) Sustainable Development Goals (SDGs).^3^, ^4^

**Figure 1:**
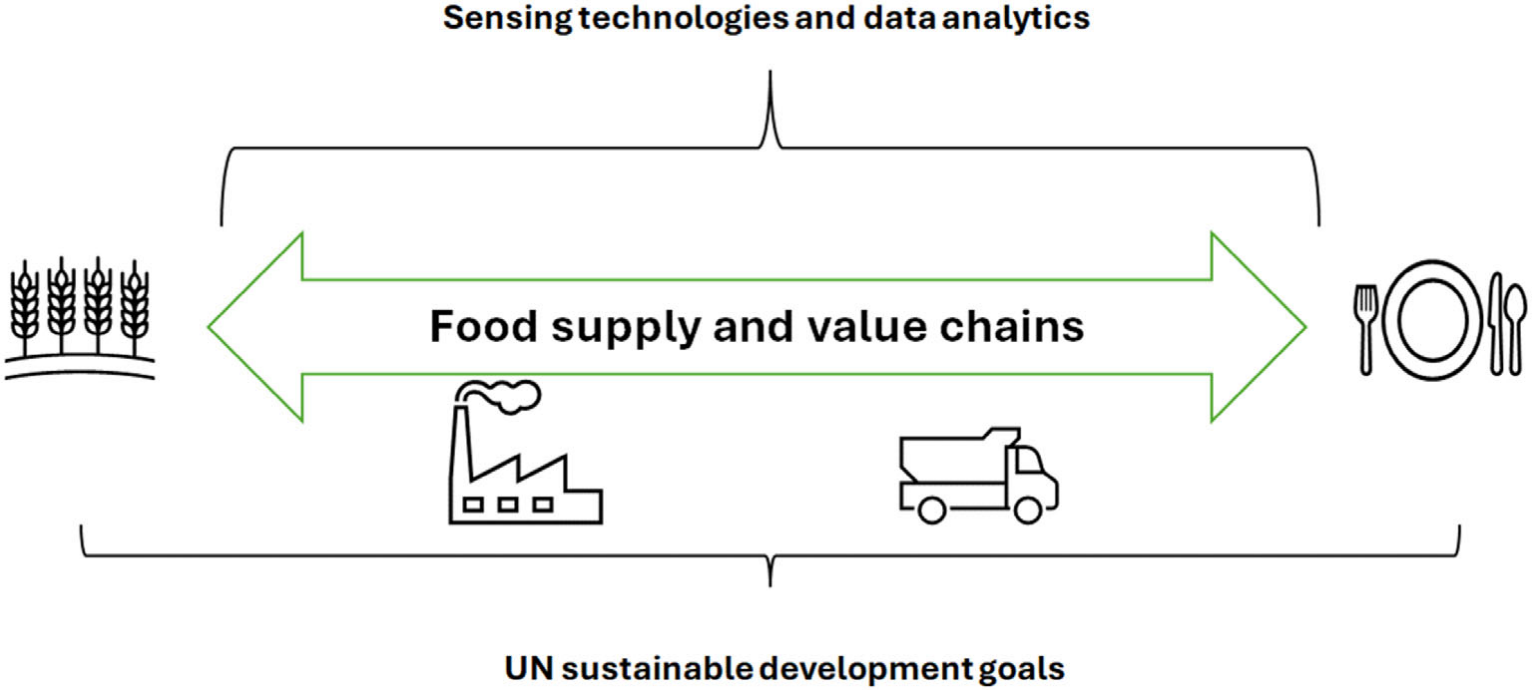
The food supply and value chains (from fork to plate) and the link with sensing technologies, data analytics and the United Nations (UN) Sustainable Development Goals (SDGs).

In conjunction with such technological advances other disruptions resulting from climate change (e.g., rising temperature and carbon dioxide (CO_2_) levels), demographics, and pandemics have also increased the pressure on agriculture as well as the reliability of food systems.^5^, ^6^ Recent data and reports emphasize the need to double the production of food by 2050 to meet the growing demands of the population.^3^, ^4^ Alongside, it is estimated that more than 30% of all food produced (approximately 930 million tonnes) is either lost or wasted throughout the agri‐food supply and value chains, resulting up to 800 million people suffering from hunger or some form of malnutrition.^5^, ^6^ Due to the prevalence of disruptions and uncertainties (e.g., climate change, new technologies, AI) concomitantly with rapid technological changes, the utilization of digital technologies is considered of particular importance to assure resilient and sustainable food. These technological advances are also contributing to guarantee food safety and security along the food supply and value chains (see Fig. 1).^7^, ^8^

The relationships between digital transformation, as one of the components of the digital economy and society index (DESI), with the UN SDGs that are directly linked with food has been evaluated by different authors.^4^, ^9^ Specifically, this relationship has been reported to enable SDG goals such as SDG1 (no poverty), SDG2 (zero hunger), SDG3 (good health and wellbeing), SDG 8 (decent work and economic growth), SDG11 (sustainable cities and communities) and SDG12 (responsible consumption and production).^4^, ^9^

Concomitantly, the associated cost of the instrumentation and implementation of these technologies has decreased dramatically with the development of on‐line and in‐line applications, as well as the availability of low‐cost hand‐held sensing spectrophotometers. These technologies have now become economically viable for their implementation within the food value chain, not only for developed, but also for developing economies.

This article provides an overview of the contributions of sensing technologies based on the utilization of vibrational spectroscopy (e.g., near‐infrared (NIR) and mid‐infrared (MIR) spectroscopy, hyperspectral imaging) combined with data analytics to successfully achieve the UN SDGs.

## THE UNITED NATIONS (UN) SUSTAINABLE DEVELOPMENT GOALS (SDGs)

The UN SDGs were defined to improve the quality of life of the global population particularly regarding social and economic aspects, with a major focus on environmental sustainability.^3^, ^4^ As described earlier, sensing and data‐driven technologies are influencing our daily lives as they have triggered changes in the economy and society.^3^, ^4^ Different studies have provided evidence on how data‐driven approaches have enabled or inhibited the successful achievement of the UN SDGs.^4^ These studies have shown that both data‐driven analytics and tools are contributing to achieve most of the UN SDGs (e.g., information becomes more reliable, data supports better‐informed decision‐making process, etc.).^4^

## FROM MEASUREMENTS TO MANAGEMENT

Issues related with food safety, quality, authenticity, provenance and fraud involve not only assessing, detecting and monitoring the prevalence of these issues but also contribute to the creation of mechanisms that allow for the rapid adaptation in and recovery of the supply and value chains.^6^, ^10^, ^11^ Therefore, the integration of sensors and data driven technologies will contribute to develop systems based on the incorporation of digital technologies to address the complexity and fragility of the food systems.^6^, ^10^ Furthermore, digital technologies will contribute through the development of robust and sustainable approaches that target food security.^12^

It is well accepted that if we want to control or manage something, we need to measure or be provided with data and information that will guide our inputs/decisions. The incorporation of digital technologies into the agri‐food sectors has become a key enabler in increasing the efficiency, productivity, and sustainability of the agri‐food systems. Digital technology and innovations that include artificial intelligence (AI), robotics, in‐ground and remote sensors, connectivity, and internet of things (IoT) are being evaluated and utilized along the whole food supply and value chains.^1^, ^2^, ^13^ The utilization of these technologies has shown their benefits in a wide range of applications including their utilization during food process optimization, monitoring food losses and waste, and overall contributing with the development of more sustainable agriculture and food systems.^1^, ^2^, ^11^

In addition, multiple researchers in the field have highlighted and discussed how the digital economy is influencing the sustainability and development of the modern food system^1^, ^2^, ^14^ as well as providing the foundations to create appropriate policies and strategies.^1^, ^14^

## SENSING TECHNOLOGIES AND DATA ANALYTICS

### Vibrational spectroscopy

Infrared (IR), both NIR and MIR, and Raman spectroscopy (RS) all utilize vibrational spectroscopy,^15^, ^16^, ^17^, ^18^ the ability to measure molecular structures as well as to identify specific molecular species in the sample are characteristics of these techniques.^15^, ^16^, ^17^, ^18^ Although, these techniques acquire the vibrational state of a given molecule or group of molecules, as not all the vibrational modes and bonds existing in a molecule are equally detected by the individual techniques, they can be combined to obtain complementary and confirmatory information from a given food sample.^16^, ^18^

Developments in MIR spectroscopy combined with Fourier‐transform (FT) has provided analysts/researchers with an instrument capable of analysing different molecule types in a wide range of samples (e.g., liquids, powders, pastes) including complex matrices such as food.^19^ The integration of attenuated total reflectance (ATR) modules with MIR instruments have further increased the analytical capability of MIR spectroscopy, particularly within the food industry.^19^ The primary advantage of the ATR module is the removal or significant reduction of sample preparation required, which allows for almost instant analysis of multiple types of samples and food matrices such as liquids, gels, powders and solids.^19^

Historically NIR spectroscopy is the most widely utilized in food analysis.^18^, ^20^ NIR spectroscopy collects the absorption energy of chemical bonds within functional groups including C–H, O–H, N–H, and C=O. These bonds are primarily related to the chemical composition and structure of foods.^16^, ^18^, ^20^ The versatility of NIR spectroscopy is associated to its ability to analyse different types of samples, including samples with high moisture content, powders, whole fruits and grains, single kernel or seeds, whole or parts of plants, among others. Furthermore, NIR spectroscopy has been implemented to analyse different type of samples in the farm, throughout harvest at‐, on‐, in‐line applications, during transport and storage of foods, and even at the supermarket.^16^, ^18^, ^20^

Improvements in instrumentation such as portability and miniaturization have shown the ability of RS in the analysis of foods.^21^, ^22^ The Raman spectrum is created by the detection of vibrations originated from changes in the electrical polarizability of molecules.^21^, ^22^ In RS, bonds that link two similar or nearly similar portions of a molecule (e.g., C–C), tend to be more active than a weakly polarizable bond (e.g., O–H).^21^, ^22^ Furthermore, RS collects information from functional groups having large polarizability (C=Cl, C=C, and C=N) in contrast to the strong absorbance bands of functional groups with strong polarization (O–H and C=O) in the IR region.^21^, ^22^ The Raman spectrum is derived from the vibrational modes of molecules originated in the IR region, where the stretching modes of these molecules appear more intense in the Raman region.^21^, ^22^ Different ranges of sample presentation methods are currently available including coherent anti‐Stokes Raman scattering, resonance RS, and surface‐enhanced Raman spectroscopy (SERS).^21^, ^22^ The main advantages of RS are the analysis of foods with high moisture content, as water is practically ‘invisible’ in the Raman spectrum compared with the IR spectrum. Conversely, interferences due to fluorescence and other instrumentation issues are still one of the major limitations of RS.^21^, ^22^

Classical NIR and MIR spectroscopy offers point based spectral information, where no spatial information is collected during the analysis,^23^, ^24^, ^25^ whereas hyperspectral imaging provides the ability of collecting both a digital image together with the molecular information of a given sample.^23^, ^24^, ^25^ Hyperspectral imaging is defined as the blend between point based spectral analysis and spatial information allowing for the assessment of the chemical composition and safety inspection of food.^23^, ^24^, ^25^ Hyperspectral imaging can utilize different wavelengths in the electromagnetic spectrum including the visible and NIR ranges, or by combining with RS.^23^, ^24^, ^25^ Despite the advantages of hyperspectral imaging, several major barriers to the widespread adoption of this technology in the food industry include the high cost of the systems available, the limited number of instrument suppliers, the prolonged time required to acquire appropriate/useful images,^23^, ^24^, ^25^, ^26^ sample preparation costs (e.g., time, resources and consumables) and the collection and processing requirements associated with image capture and analysis.^23^, ^24^, ^25^

The terahertz (THz) region is located between the end of the IR region and the start of the microwave region.^27^ Although, the utilization of THz in food analysis is still considered in its infancy stages, this technique offers the possibility to characterize the far IR vibrational modes in food.^27^ The advantage of THz spectroscopy has been its ability to measure water in diverse food matrices due to THz waves overlap with the vibration of the hydrogen bond. This region is well known to be highly sensitive to the presence of water.^27^

Recently, the combination of vibrational spectroscopy with optical microscopy (e.g., confocal and optical microscope) has provided the user the ability to collect images from a wide range of food matrices (e.g., cereals, single kernel; animal muscles),^22^ moreover, the combination of optical microscopy with vibrational spectroscopy allows for the analysis of the chemical composition of food samples without the need for staining or extensive sample preparation.^22^

### Data analytics

The practical application and implementation of vibrational spectroscopy in food safety and quality requires the utilization of data analytics, including chemometrics and machine learning (ML) techniques.^28^, ^29^ For example, the determination of authenticity or the level of fraud in a food using classification methods is based on the utilization of data as input (e.g., absorbance values at specific wavelengths or frequencies) where the outputs or results can be the classes (classification process) defined during the training or calibration step.^28^, ^29^ Furthermore, these clusters, groups, classes or patterns can be predicted from new input data collected. Overall, the ability of the models to predict or classify new samples will depend on the quality of the data collected (e.g., wavelength range, signal to noise ratio, sample presentation).^28^, ^29^

During the implementation of vibrational spectroscopy techniques, two type of methodologies can be utilized to analyse the data generated, untargeted or targeted analysis.^30^, ^31^ Targeted methods are founded on the quantification of known characteristics, ingredients or properties in the sample.^30^, ^31^ However, untargeted analysis is founded on the collection and interpretation of signals obtained from the application of different methods or techniques.^30^, ^31^

The analysis of the data determines the selection of different algorithms for later application. These algorithms or techniques have provided the basis for the different types of analysis (e.g., targeted and untargeted analysis, calibration development, classification).^30^, ^31^, ^32^ Examples of these algorithms include principal component analysis (PCA), partial least squares regression (PLS), support vector machines (SVMs) classification or regression, artificial neural networks (ANNs), K‐Nearest neighbours (KNNs), and convolutional neural networks (CNNs).^30^, ^31^, ^32^

Recent applications of food fingerprinting have reported the utilization of the so‐called data fusion methods for food analysis.^33^ Data fusion has shown promising results during the development of applications that combines data from different platforms or instruments.^33^ Although, this methodology seems to be straightforward, it still presents an important challenge in the field of data analytics in food applications of chemometrics.^33^ Developments in sensing technologies also encompasses the incorporation of different methods and algorithms that have been used to analyse the data generated during the application of sensors, the utilization of pre‐processing (e.g., baseline, derivatives) is required when calibration and classification models are developed.^34^, ^35^

## EXAMPLES OF VIBRATIONAL SPECTROSCOPY AND DATA ANALYTICS TO ACHIEVE THE UN SDG TARGETS

### Food safety (SDG2, no hunger; SDG3, good health)

Food safety and quality are two of the fields where sensing technologies and data analytics have had a great impact in recent years.^36^, ^37^, ^38^, ^39^, ^40^ Food safety is considered as a system that integrates appropriate food production, handling, processing, and trading with the aim to have negligible to zero probability of contracting a foodborne disease.^36^, ^37^, ^38^, ^39^, ^40^ It is well known that food safety hazards can arise at any stage of the food system from farm to fork.^36^, ^37^, ^38^, ^39^, ^40^ Food safety is directly linked with microbiological hazards associated with the presence of living microorganisms.^36^, ^37^, ^38^, ^39^, ^40^ The presence of these microorganisms could determine food spoilage and in severe cases, food poisoning.^36^, ^37^, ^38^, ^39^, ^40^ In addition to the presence of microorganisms, other hazards such as chemical (e.g., chemicals utilized in the agricultural production and food processing), toxic compounds (e.g., mycotoxins, biotoxins, and environmental contaminants); and technological hazards (e.g., food irradiation and genetic modification of food) are also frequently encountered.^36^, ^37^, ^38^, ^39^, ^40^ To control and monitor food safety hazards as well as to demonstrate compliance with food safety standards (e.g., HACCP (hazard analysis and critical control points), ISO 22000, the different players along the food production chain must monitor the key safety indicators at the critical points to keep transparent and reliable records regarding performed measurements.^36^, ^37^, ^38^, ^39^, ^40^ Monitoring these indicators using traditional (wet chemistry) methods are cumbersome, expensive, time consuming and in most cases, require the destruction of the sample being analysed. The utilization and incorporation of technology and the development of new methods to identify, monitor, as well as to assess food safety hazards, that are also coupled with data analytics are now being incorporated by analytical and quality laboratories as well as by the food manufacturing industry to circumvent these shortcomings of the traditional analytical technologies.^36^, ^41^

Several examples on the utilization of AI and ML combined with sensing technologies to achieve the UN SDG targets have been developed.^40^ The utilization of sensing technologies has provided the means to develop efficient management systems to address issues associated with safety at the farm level (e.g., contaminants, presence of pesticides) as well as to monitor food safety along the supply and value chains (e.g., microorganism, animal toxins, mycotoxins).^36^, ^42^ The utilization of sensing technologies also provide both data and the information to improve existing or to develop novel safety management systems thereby preventing numerous issues associated with safety.^36^, ^42^

### Food quality, composition and nutritional value (SDG2, no hunger; SDG3, good health; SDG12, responsible consumption)

In addition to food safety, food quality has been defined as the totality of the different properties and assessable attributes of a food ingredient or products.^43^ Different biochemical, chemical and physical characteristics or properties are utilized to define food quality including colour, flavour, texture, taste, nutritional value, functional properties and chemical composition.^43^ Recently, food provenance, or issues associated with animal welfare, environmentally friendly production, and sustainable farming practices, have become attributes and properties that are important to contribute to a holistic definition of food quality.^43^ Furthermore, food quality not only has the objective definition as described earlier but also a subjective component, as perceived by consumers.^43^ It is in this field that sensing and data driven technologies have contributed the most where a large number of examples can be found in the literature on the applications of vibrational spectroscopy to predict the chemical composition and nutritive value of food ingredients and products^43^ such as cheese,^44^ fish,^46^ meat burgers/patties,^45^ horticultural products and fruits,^47^, ^48^ agricultural products,^49^, ^50^ bioactive compounds in a wide range of plant derived foods.^51^ Hyperspectral imaging has been also evaluated as a tool to determine the quality of food ingredients and products^52^ and has been shown to be efficient after the application of different pre‐processing methods.^26^

### Food authenticity, provenance and fraud (SDG3, good health; SDG12, responsible consumption)

In addition to food safety and quality, food authenticity (e.g., provenance, geographical origin) and fraud have become very important to both consumer and the food industry.^40^, ^53^ Assessing and monitoring food authenticity and fraud involves the utilization of new ways of analysis where the utilization of sensing technologies has been proven to be in the front line of analysis as demonstrated by different reports.^54^, ^55^, ^56^, ^57^ Although, a wide range of analytical techniques are utilized to assure and monitor food authenticity and fraud, vibrational spectroscopy (e.g., NIR, MIR and Raman) is considered one of the most utilized techniques. The scientific literature has shown a plethora of examples in this field.^58^, ^59^, ^60^, ^61^, ^62^ Foods of great economic value are the ones that are usually subjected to food adulteration or fraud. They include premium grains,^63^, ^64^ honey,^65^ spices and herbs (e.g., saffron, oregano, pepper),^66^, ^67^ meat,^68^, ^69^, ^70^, ^71^, ^72^ milk and dairy products,^73^ olive oil,^74^, ^75^ coffee,^76^, ^77^, ^78^, ^79^ beer and wine,^79^, ^80^, ^81^ fish and seafood.^82^ Overall, each of these foods present with their unique technical challenges to verify their authenticity.

### Food optimization and process control (SDG9, innovation and infrastructure)

The optimization and monitoring of the composition and safety of food ingredients, and products, are still based on the utilization of discontinuous analyses. In this space, traditional analytical laboratory methods have been used during the quality control of foods where most of these laboratories are far from the production line.^83^, ^84^ However, this approach is no longer satisfactory to fulfil the needs of the modern food industry and the SDG where increases in efficiency and productivity for augmented sustainability are paramount.^84^, ^85^, ^86^ Modern food production systems are required to maintain higher safety and quality standards in parallel with demands in high throughput data and information at the production facilities, due to an increase in the number of samples to be analysed.^83^, ^84^ Sensing technologies have been essential to the development of process analytical technologies (PATs). The PAT approach has been implemented by the food industry to address the need for increased efficiency during production and has been implemented along the different steps of the supply, and value chains. The PAT has provided stakeholders/producers/analysts with a better understanding and control of the raw materials, intermediate products during the production process, packing and delivery of foods.^83^, ^84^, ^85^, ^86^ Overall, the implementation of sensing technologies together with PATs has provided a real time analysis of the food process, allowing for a better monitoring of the food process, avoiding waste and increasing efficiencies during the processing of food products.^83^, ^84^, ^85^, ^86^ Process analytics have become essential to increase efficiency and sustainability of the processing. The use of IR spectroscopy can support decision management systems during the evaluation of the most suitable processing methods and control systems throughout the quality control of different foods. Examples from research and the food manufacturing industry are providing evidence of the utilization of sensing technologies applied to monitor the processing of foods (e.g., at‐, on‐ and in‐line applications) in milk and dairy products^87^; meat and meat products^88^, ^89^ and during food fermentation.^85^, ^86^ Table 1 summarizes applications of sensing technologies and data analytics and the link with the UN SDG.

**Table 1:** Applications of sensing technologies and data analytics and the link with the United Nations (UN) Sustainable Development Goals (SDGs)

Application	Link with UN SDG	Observations
Microorganism, other hazards such as chemical (e.g., chemicals utilized in the agricultural and food processing), toxic compounds (e.g., mycotoxins, biotoxins, and environmental contaminants); and technological hazards (e.g., food irradiation and genetic modification of food)	SDG3 = good health	Monitoring and detecting microorganisms, toxins is contributing to better health and assure the safety of foods consume
Food composition, nutritional value (e.g., crude protein, lipid and fat content, starch, amino acids, etc.)	SDG2 = no hunger; SDG3 = good health	Sensing technologies and data analytics are used to measure and assess the composition and nutritional value of different agricultural commodities, plant and animal products
Bioactive compounds and functionality (e.g., anthocyanins, phenolic compounds, pasting properties, etc.)	SDG3 = good health	The measurement and prediction of bio compounds and functional properties of food ingredients and products to promote good health
Authenticity, provenance, fraud	SDG2 = no hunger; SDG3 = good health; SDG12 = responsible consumption	The control and analysis of the provenance, authenticity of premium foods and ingredients, the assessment of fraud contribute to the safety of the foods, promoting health, and responsible consumption depending on the region of production, etc.
Process control, optimization, monitoring	SDG9 = innovation and infrastructure	The ability to control and monitor a process improves the efficiency and productivity of food production

## CHALLENGES FACING THE IMPLEMENTATION OF SENSING TECHNOLOGIES AND DATA ANALYTICS

The increasing utilization of digital technologies such as vibrational spectroscopy and data analytics in the food supply and value chains, has provided several opportunities as well as challenges.^90^ Furthermore, the utilization of digital technologies is fostering the development of new strategies or management systems to address issues associated with food safety and quality.^54^, ^55^, ^91^, ^92^

Visualization of the data, as well as issues associated with data creation and collection, data curation, and data analysis have become part of the analysis. Unfortunately, these challenges can hinder the adoption of digital technologies and data driven analytics by the food manufacturing industry, regulatory authorities, and routine quality control laboratories. Implementing sensing technologies and data analytics in addressing food safety, quality and fraud can be hindered by the lack of training or education. This training is not only required at the industry level but also at universities. Technical issues such as the selection of the appropriate data analysis methods (e.g., ML), challenges associated with sampling (e.g., where and how to collect a representative sample), the selection of the appropriate instrument or sensor, are still not well defined or understood.

Traditional methodologies or analytical approaches currently in use are comparatively more expensive, time‐consuming, and require highly specialized instrumentation and skilled operators.^54^, ^55^ Consequently, the implementation of sensing technologies where less consumables and less time in sample preparation is required, have become more attractive as they can be a more time and cost‐effective analysis per sample.

It is a given that the use of digital and sensing technologies to assess and monitor foods along the supply and value chains is becoming important to assure compliance with quality control and regulatory systems.^16^, ^18^, ^57^, ^92^, ^93^ For example, testing agricultural commodities and food at the collection point (e.g., distribution centres, market) will reduce the potential deterioration and damage to the food during transportation to the more traditional analytical or quality control laboratories. Moreover, the advent of sensing technologies eliminates the need for expensive and time‐consuming analyses utilized along the food supply and value chains to guarantee safety and quality.

These technologies are highly dependent on operator expertise as obtaining reliable results with high accuracy requires specialized knowledge. In this respect providing training for individuals in the food supply and value chain will be useful. Current developments in portable devices have provided not only new tools that are capable of collecting multi‐dimensional and complex data but also new challenges. The integration of data transfer systems into different devices would enable the combination of sensors with remote data storage (e.g., cloud) and consequently maintain records along the supply and value chains.^94^ It is recognized that the utilization of sensing technologies has proven useful for monitoring most of the different steps of the supply and value chains (from gate to plate). The incorporation of sensing technologies, IoT, blockchain, throughout the entire food value chain provides numerous benefits, such as increasing productivity, safety, consumer confidence, enhancing the transparency of the food systems.

However, new challenges that arise from the use of these technologies is the storage of the large data collected as well as ensuring the integrity (security and privacy) of the data.^94^ Cyber security and data science researchers have started constructing advanced technologies to safeguard the information, one such technology is the so‐called smart agriculture testbed (SATB) as described by Wan *et al*.^13^ Their test bed was designed to capture data that covers the whole life cycle of the smart agriculture scenario from the sensors incorporated during the production/processing cycles as well as incorporating protocols that include authentication and that can also generate reports on attack traces of the networks and vulnerability scanning. These aspects are of particular importance to the global trade in various food commodities to ensure that the global sustainability goals are met.

## CONCLUSIONS

The integration of technology and data analytics into the food supply and value chain is undoubtably required to ensure efficient and reliable measurement of food safety and quality. However, there is a need to better understand the background of sensing technologies to ensure accurate interpretation of the different data processing and analytics tools and ultimately the results and the application. The research community and the food manufacturing industry have embraced in a dissimilar way the utilization of sensing technologies to monitor food safety, quality and different steps during the processing and value chain. One of the limitations is to what extent the utilization of sensors combined with data analytics and the results derived from their application, will be repeatable and comparable to those obtained using traditional biological, biochemical and chemical methods (this of course assumes that these traditional methods are indeed the golden standard). The validation or verification of the results will be of principal importance in judging the reliability of these technologies to assess food safety and quality, as well as ensuring public and authority acceptance thereof.

The implementation of digital technologies (e.g., sensors, data analyses) requires close collaboration between researchers, the food manufacturing industries, consumers and cyber security experts. This process requires a systems approach that should incorporate multidisciplinary teams. This multidisciplinary and inclusive approach will be of importance to apply digital technologies and to better understand food systems. The incorporation of technology will provide tools that can be used to monitor and predict the safety and quality of foods. Furthermore, digital technologies are enabling the development of novel decision‐management systems along the food supply and value chain. Ultimately, the goal will be to assure the consumers the use of these technologies plays a key role in the applicability of the UN SDG.

## FUNDING

No external funding.

## Data Availability

Data sharing not applicable to this article as no datasets were generated or analysed during the current study.
